# 2-[2-(2-Carb­oxy­phen­yl)hydrazinyl­idene]-3-oxo-*N*-phenyl­butyramide

**DOI:** 10.1107/S1600536811048173

**Published:** 2011-11-23

**Authors:** Jinlong Dong, Gailing Zhang, Meiyu Guo, Chuan Wu, Jianguo Ren

**Affiliations:** a Department of Chemistry, Taiyuan Normal University, Taiyuan 030031, People’s Republic of China; b School of Chemistry and Chemical Engineering, Shanxi University, Taiyuan 030006, People’s Republic of China

## Abstract

In the title compound, C_17_H_15_N_3_O_4_, the mol­ecule is in the keto–hydrazone form. Intra­molecular N—H⋯O hydrogen bonds ensure that the mol­ecule is nearly planar (r.m.s. deviation of non-H atoms is 0.098 Å), with the two benzene rings forming a dihedral angle of 10.04 (2)°. In the crystal, inversion dimers are formed *via* pairs of O—H⋯O hydrogen bonds involving the –CO_2_H groups.

## Related literature

For general background to the properties of organic pigments, see: Schmidt *et al.* (2007 ▶); Barrow *et al.* (2002 ▶). For related structures, see: van de Streek *et al.* (2009 ▶). For standard bond-length data, see: Allen *et al.* (1987 ▶).
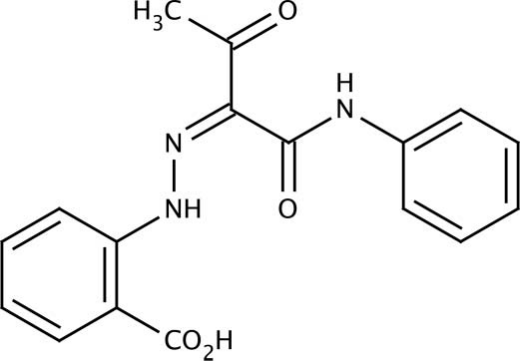


## Experimental

### 

#### Crystal data

C_17_H_15_N_3_O_4_
*M* *_r_* = 325.32
Monoclinic, 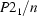

*a* = 15.5731 (16) Å
*b* = 5.3292 (5) Å
*c* = 18.7731 (19) Å
β = 99.246 (1)°
*V* = 1537.8 (3) Å^3^
*Z* = 4
Mo *K*α radiation
μ = 0.10 mm^−1^
*T* = 298 K
0.50 × 0.30 × 0.21 mm

#### Data collection

Bruker SMART CCD area-detector diffractometer
Absorption correction: multi-scan (*SADABS*; Bruker, 2007 ▶) *T*_min_ = 0.951, *T*_max_ = 0.979
7277 measured reflections
2724 independent reflections
1459 reflections with *I* > 2σ(*I*)
*R*_int_ = 0.057

#### Refinement

*R*[*F* ^2^ > 2σ(*F* ^2^)] = 0.048
*wR*(*F* ^2^) = 0.137
*S* = 1.02
2724 reflections
219 parameters
H-atom parameters constrained
Δρ_max_ = 0.19 e Å^−3^
Δρ_min_ = −0.17 e Å^−3^

### 

Data collection: *SMART* (Bruker, 2007 ▶); cell refinement: *SAINT* (Bruker, 2007 ▶); data reduction: *SAINT*; program(s) used to solve structure: *SHELXS97* (Sheldrick, 2008 ▶); program(s) used to refine structure: *SHELXL97* (Sheldrick, 2008 ▶); molecular graphics: *SHELXTL* (Sheldrick, 2008 ▶); software used to prepare material for publication: *SHELXTL*.

## Supplementary Material

Crystal structure: contains datablock(s) I, global. DOI: 10.1107/S1600536811048173/pk2357sup1.cif

Structure factors: contains datablock(s) I. DOI: 10.1107/S1600536811048173/pk2357Isup2.hkl

Supplementary material file. DOI: 10.1107/S1600536811048173/pk2357Isup3.cml

Additional supplementary materials:  crystallographic information; 3D view; checkCIF report

## Figures and Tables

**Table 1:** Hydrogen-bond geometry (Å, °)

*D*—H⋯*A*	*D*—H	H⋯*A*	*D*⋯*A*	*D*—H⋯*A*
O4—H4⋯O3^i^	0.82	1.84	2.654 (3)	175
N3—H3⋯O1	0.86	1.97	2.685 (3)	140
N1—H1⋯O3	0.86	1.99	2.631 (3)	131
N1—H1⋯O2	0.86	1.91	2.568 (3)	132

Symmetry code: (i) 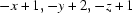.

**Fig. 1.**
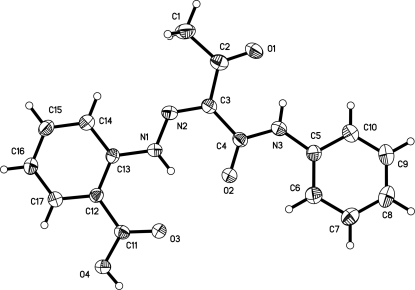
The molecular structure of the title compound with displacement ellipsoids drawn at the 30% probability level.

**Fig. 2.**
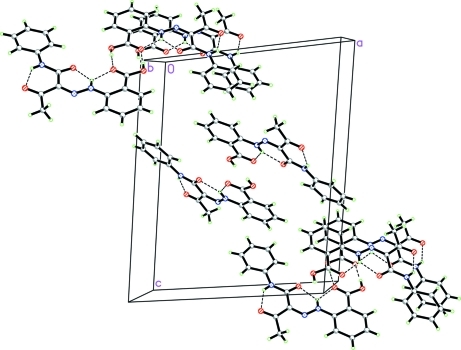
Part of the crystal structure of (I) viewed approximately down the b axis, with hydrogen bonds drawn as dashed lines.

## supplementary crystallographic information

### Comment

Organic pigments are nowadays most commonly used for coloring paints and plastics and for most printing applications (Schmidt *et al.*, 2007; Barrow *et al.*, 2002). Originally, azo pigments were believed to contain the azo group N═N, but for approximately 25 years now it has been known that all Hansa yellow pigments (and all other commercial 'azo' pigments) crystallize in the hydrazone form; it would, therefore, be more appropriate to speak of 'hydrazone' pigments (van de Streek *et al.*, 2009). In this paper, we present the crystal structure of the title hydrazone compound (I). The molecular structure of (I) is shown in Fig. 1. The bond lengths (Allen *et al.*, 1987) and angles are normal. The O2—C4 (1.226 (3) Å) and N3—C4 (1.343 (3) Å) distances are consistent with the keto form of the amide functionality. The N1—N2 (1.310 (3) Å) and N2—C3(1.306 (3) Å) distances are consistent with hydrazone form functionality.

### Experimental

To a sodium hydroxide solution (20 cm^3^) of acetoacetanilide (102 mmol), 40 cm^3^ (101 mmol) diazcompound of *o*-aminobenzoic acid was added and the mixture was heated to 90° for 1 h. A yellow solid separated and was collected by filtration, washed with diethyl ether (absolute) and dried in air. Then the precipitate was dissolved in methanol and the solution was allowed to stand for a few days at ambient temperature, after which time yellow blocky crystals of the title compound suitable for X-ray diffraction were obtained.

### Refinement

H atoms were placed in idealized positions and allowed to ride on their respective parent atoms, with C—H = 0.93–0.96 Å, N—H = 0.86Å and *U*_iso_(H)= 1.2*U*_eq_(C, N) or 1.5*U*_eq_(C_methyl_, O).

### Crystal data

**Table:**

C_17_H_15_N_3_O_4_	*F*(000) = 680
*M**_r_* = 325.32	*D*_x_ = 1.405 Mg m^−^^3^
Monoclinic, *P*2_1_/*n*	Mo *K*α radiation, λ = 0.71073 Å
Hall symbol: -P 2yn	Cell parameters from 1353 reflections
*a* = 15.5731 (16) Å	θ = 2.7–21.8°
*b* = 5.3292 (5) Å	µ = 0.10 mm^−^^1^
*c* = 18.7731 (19) Å	*T* = 298 K
β = 99.246 (1)°	Prism, yellow
*V* = 1537.8 (3) Å^3^	0.50 × 0.30 × 0.21 mm
*Z* = 4	

### Data collection

**Table:**

Bruker SMART CCD area-detector diffractometer	2724 independent reflections
Radiation source: fine-focus sealed tube	1459 reflections with *I* > 2σ(*I*)
graphite	*R*_int_ = 0.057
φ and ω scans	θ_max_ = 25.0°, θ_min_ = 2.7°
Absorption correction: multi-scan (*SADABS*; Bruker, 2007)	*h* = −18→17
*T*_min_ = 0.951, *T*_max_ = 0.979	*k* = −6→6
7277 measured reflections	*l* = −16→22

### Refinement

**Table:**

Refinement on *F*^2^	Primary atom site location: structure-invariant direct methods
Least-squares matrix: full	Secondary atom site location: difference Fourier map
*R*[*F*^2^ > 2σ(*F*^2^)] = 0.048	Hydrogen site location: inferred from neighbouring sites
*wR*(*F*^2^) = 0.137	H-atom parameters constrained
*S* = 1.02	*w* = 1/[σ^2^(*F*_o_^2^) + (0.044*P*)^2^ + 0.5365*P*] where *P* = (*F*_o_^2^ + 2*F*_c_^2^)/3
2724 reflections	(Δ/σ)_max_ < 0.001
219 parameters	Δρ_max_ = 0.19 e Å^−^^3^
0 restraints	Δρ_min_ = −0.17 e Å^−^^3^

### Special details

**Table:**

Geometry. All e.s.d.'s (except the e.s.d. in the dihedral angle between two l.s. planes) are estimated using the full covariance matrix. The cell e.s.d.'s are taken into account individually in the estimation of e.s.d.'s in distances, angles and torsion angles; correlations between e.s.d.'s in cell parameters are only used when they are defined by crystal symmetry. An approximate (isotropic) treatment of cell e.s.d.'s is used for estimating e.s.d.'s involving l.s. planes.
Refinement. Refinement of *F*^2^ against ALL reflections. The weighted *R*-factor *wR* and goodness of fit *S* are based on *F*^2^, conventional *R*-factors *R* are based on *F*, with *F* set to zero for negative *F*^2^. The threshold expression of *F*^2^ > 2σ(*F*^2^) is used only for calculating *R*-factors(gt) *etc*. and is not relevant to the choice of reflections for refinement. *R*-factors based on *F*^2^ are statistically about twice as large as those based on *F*, and *R*- factors based on ALL data will be even larger.

### Fractional atomic coordinates and isotropic or equivalent isotropic displacement parameters (Å^2^)

**Table:**

	*x*	*y*	*z*	*U*_iso_*/*U*_eq_	
N1	0.41408 (14)	0.3850 (5)	0.60735 (12)	0.0434 (7)	
H1	0.3974	0.5025	0.5769	0.052*	
N2	0.35836 (15)	0.2176 (4)	0.62294 (12)	0.0419 (6)	
N3	0.16263 (15)	0.3518 (5)	0.49622 (13)	0.0505 (7)	
H3	0.1377	0.2185	0.5086	0.061*	
O1	0.15219 (14)	−0.0370 (4)	0.58537 (12)	0.0645 (7)	
O2	0.28838 (12)	0.5712 (4)	0.51762 (11)	0.0555 (6)	
O3	0.46446 (12)	0.7749 (4)	0.53863 (11)	0.0549 (6)	
O4	0.60410 (12)	0.8767 (4)	0.55453 (10)	0.0512 (6)	
H4	0.5857	0.9845	0.5248	0.077*	
C1	0.2678 (2)	−0.1513 (6)	0.67626 (18)	0.0644 (10)	
H1A	0.2247	−0.2606	0.6908	0.097*	
H1B	0.2911	−0.0448	0.7159	0.097*	
H1C	0.3137	−0.2498	0.6620	0.097*	
C2	0.2268 (2)	0.0067 (6)	0.61396 (17)	0.0493 (8)	
C3	0.27837 (17)	0.2113 (6)	0.58885 (15)	0.0412 (7)	
C4	0.24362 (18)	0.3937 (6)	0.53112 (15)	0.0415 (8)	
C5	0.11349 (18)	0.4973 (6)	0.44218 (15)	0.0439 (8)	
C6	0.1443 (2)	0.7084 (6)	0.41191 (17)	0.0520 (9)	
H6	0.2013	0.7612	0.4265	0.062*	
C7	0.0899 (2)	0.8403 (6)	0.35986 (17)	0.0578 (9)	
H7	0.1107	0.9828	0.3396	0.069*	
C8	0.0057 (2)	0.7644 (7)	0.33746 (18)	0.0640 (10)	
H8	−0.0305	0.8554	0.3025	0.077*	
C9	−0.0247 (2)	0.5538 (7)	0.36703 (18)	0.0614 (10)	
H9	−0.0818	0.5017	0.3521	0.074*	
C10	0.02861 (19)	0.4196 (6)	0.41859 (17)	0.0534 (9)	
H10	0.0078	0.2755	0.4379	0.064*	
C11	0.53920 (18)	0.7376 (5)	0.56816 (15)	0.0401 (7)	
C12	0.56303 (17)	0.5356 (5)	0.62085 (14)	0.0375 (7)	
C13	0.50074 (17)	0.3708 (6)	0.64061 (14)	0.0375 (7)	
C14	0.52611 (19)	0.1898 (6)	0.69253 (15)	0.0471 (8)	
H14	0.4849	0.0824	0.7067	0.057*	
C15	0.6116 (2)	0.1679 (6)	0.72323 (16)	0.0514 (9)	
H15	0.6280	0.0467	0.7584	0.062*	
C16	0.6735 (2)	0.3229 (6)	0.70263 (16)	0.0531 (9)	
H16	0.7317	0.3030	0.7225	0.064*	
C17	0.64912 (18)	0.5063 (6)	0.65278 (15)	0.0462 (8)	
H17	0.6911	0.6139	0.6400	0.055*	

### Atomic displacement parameters (Å^2^)

**Table:**

	*U*^11^	*U*^22^	*U*^33^	*U*^12^	*U*^13^	*U*^23^
N1	0.0420 (14)	0.0388 (16)	0.0482 (16)	−0.0049 (12)	0.0037 (12)	0.0094 (12)
N2	0.0447 (14)	0.0366 (16)	0.0454 (15)	−0.0032 (12)	0.0106 (12)	0.0008 (12)
N3	0.0444 (15)	0.0445 (17)	0.0609 (18)	−0.0091 (13)	0.0035 (13)	0.0065 (14)
O1	0.0483 (13)	0.0586 (16)	0.0874 (17)	−0.0120 (12)	0.0129 (12)	0.0133 (13)
O2	0.0417 (12)	0.0536 (15)	0.0688 (15)	−0.0063 (11)	0.0017 (10)	0.0184 (12)
O3	0.0425 (13)	0.0525 (15)	0.0654 (15)	−0.0061 (11)	−0.0042 (10)	0.0196 (12)
O4	0.0452 (12)	0.0473 (14)	0.0600 (14)	−0.0052 (11)	0.0054 (10)	0.0145 (11)
C1	0.080 (2)	0.052 (2)	0.062 (2)	−0.0127 (19)	0.0126 (18)	0.0109 (19)
C2	0.053 (2)	0.041 (2)	0.057 (2)	−0.0031 (17)	0.0184 (16)	−0.0053 (17)
C3	0.0392 (17)	0.0390 (19)	0.0466 (19)	−0.0019 (15)	0.0101 (14)	−0.0008 (15)
C4	0.0367 (17)	0.042 (2)	0.0473 (19)	−0.0016 (15)	0.0098 (14)	0.0003 (15)
C5	0.0417 (17)	0.046 (2)	0.0440 (18)	0.0026 (15)	0.0073 (14)	−0.0032 (16)
C6	0.0528 (19)	0.049 (2)	0.054 (2)	−0.0001 (17)	0.0078 (16)	−0.0036 (17)
C7	0.066 (2)	0.050 (2)	0.057 (2)	0.0092 (19)	0.0104 (18)	0.0040 (18)
C8	0.062 (2)	0.075 (3)	0.052 (2)	0.023 (2)	0.0026 (17)	−0.001 (2)
C9	0.0444 (19)	0.077 (3)	0.060 (2)	0.006 (2)	0.0028 (17)	−0.010 (2)
C10	0.0465 (19)	0.056 (2)	0.058 (2)	−0.0024 (17)	0.0093 (16)	−0.0066 (18)
C11	0.0435 (18)	0.0364 (19)	0.0408 (18)	−0.0064 (15)	0.0080 (14)	−0.0025 (15)
C12	0.0409 (16)	0.0365 (18)	0.0340 (16)	0.0000 (14)	0.0030 (13)	0.0001 (14)
C13	0.0386 (16)	0.0402 (19)	0.0330 (16)	0.0005 (14)	0.0030 (13)	−0.0007 (14)
C14	0.0478 (18)	0.049 (2)	0.0436 (19)	−0.0014 (16)	0.0053 (14)	0.0041 (16)
C15	0.057 (2)	0.048 (2)	0.047 (2)	0.0039 (17)	0.0000 (16)	0.0096 (17)
C16	0.0465 (19)	0.054 (2)	0.054 (2)	0.0002 (17)	−0.0056 (16)	0.0070 (18)
C17	0.0433 (17)	0.047 (2)	0.0466 (19)	−0.0033 (16)	0.0031 (14)	0.0024 (16)

### Geometric parameters (Å, °)

**Table:**

N1—N2	1.310 (3)	C6—C7	1.378 (4)
N1—C13	1.395 (3)	C6—H6	0.9300
N1—H1	0.8600	C7—C8	1.373 (4)
N2—C3	1.306 (3)	C7—H7	0.9300
N3—C4	1.343 (3)	C8—C9	1.370 (5)
N3—C5	1.402 (4)	C8—H8	0.9300
N3—H3	0.8600	C9—C10	1.371 (4)
O1—C2	1.221 (3)	C9—H9	0.9300
O2—C4	1.226 (3)	C10—H10	0.9300
O3—C11	1.222 (3)	C11—C12	1.469 (4)
O4—C11	1.311 (3)	C12—C17	1.387 (4)
O4—H4	0.8200	C12—C13	1.402 (4)
C1—C2	1.498 (4)	C13—C14	1.383 (4)
C1—H1A	0.9600	C14—C15	1.369 (4)
C1—H1B	0.9600	C14—H14	0.9300
C1—H1C	0.9600	C15—C16	1.370 (4)
C2—C3	1.476 (4)	C15—H15	0.9300
C3—C4	1.491 (4)	C16—C17	1.364 (4)
C5—C6	1.381 (4)	C16—H16	0.9300
C5—C10	1.388 (4)	C17—H17	0.9300
			
N2—N1—C13	119.4 (2)	C6—C7—H7	119.5
N2—N1—H1	120.3	C9—C8—C7	119.5 (3)
C13—N1—H1	120.3	C9—C8—H8	120.2
C3—N2—N1	121.5 (3)	C7—C8—H8	120.2
C4—N3—C5	128.4 (3)	C8—C9—C10	120.2 (3)
C4—N3—H3	115.8	C8—C9—H9	119.9
C5—N3—H3	115.8	C10—C9—H9	119.9
C11—O4—H4	109.5	C9—C10—C5	120.5 (3)
C2—C1—H1A	109.5	C9—C10—H10	119.7
C2—C1—H1B	109.5	C5—C10—H10	119.7
H1A—C1—H1B	109.5	O3—C11—O4	121.8 (3)
C2—C1—H1C	109.5	O3—C11—C12	122.9 (3)
H1A—C1—H1C	109.5	O4—C11—C12	115.3 (2)
H1B—C1—H1C	109.5	C17—C12—C13	118.5 (3)
O1—C2—C3	121.9 (3)	C17—C12—C11	119.7 (3)
O1—C2—C1	119.4 (3)	C13—C12—C11	121.8 (2)
C3—C2—C1	118.6 (3)	C14—C13—N1	120.0 (3)
N2—C3—C2	112.8 (3)	C14—C13—C12	119.5 (3)
N2—C3—C4	123.1 (3)	N1—C13—C12	120.5 (3)
C2—C3—C4	124.1 (3)	C15—C14—C13	120.3 (3)
O2—C4—N3	123.2 (3)	C15—C14—H14	119.8
O2—C4—C3	120.1 (3)	C13—C14—H14	119.8
N3—C4—C3	116.7 (3)	C14—C15—C16	120.7 (3)
C6—C5—C10	119.2 (3)	C14—C15—H15	119.7
C6—C5—N3	124.3 (3)	C16—C15—H15	119.7
C10—C5—N3	116.6 (3)	C17—C16—C15	119.7 (3)
C7—C6—C5	119.6 (3)	C17—C16—H16	120.2
C7—C6—H6	120.2	C15—C16—H16	120.2
C5—C6—H6	120.2	C16—C17—C12	121.3 (3)
C8—C7—C6	121.0 (3)	C16—C17—H17	119.3
C8—C7—H7	119.5	C12—C17—H17	119.3
			
C13—N1—N2—C3	−174.3 (3)	C8—C9—C10—C5	0.9 (5)
N1—N2—C3—C2	179.2 (2)	C6—C5—C10—C9	−1.5 (5)
N1—N2—C3—C4	−1.1 (4)	N3—C5—C10—C9	178.6 (3)
O1—C2—C3—N2	−175.6 (3)	O3—C11—C12—C17	−179.3 (3)
C1—C2—C3—N2	4.2 (4)	O4—C11—C12—C17	0.9 (4)
O1—C2—C3—C4	4.7 (5)	O3—C11—C12—C13	0.2 (4)
C1—C2—C3—C4	−175.4 (3)	O4—C11—C12—C13	−179.7 (2)
C5—N3—C4—O2	−2.5 (5)	N2—N1—C13—C14	−3.6 (4)
C5—N3—C4—C3	177.4 (3)	N2—N1—C13—C12	175.1 (2)
N2—C3—C4—O2	−5.9 (4)	C17—C12—C13—C14	1.9 (4)
C2—C3—C4—O2	173.7 (3)	C11—C12—C13—C14	−177.5 (3)
N2—C3—C4—N3	174.3 (3)	C17—C12—C13—N1	−176.8 (3)
C2—C3—C4—N3	−6.1 (4)	C11—C12—C13—N1	3.7 (4)
C4—N3—C5—C6	4.3 (5)	N1—C13—C14—C15	177.3 (3)
C4—N3—C5—C10	−175.7 (3)	C12—C13—C14—C15	−1.5 (4)
C10—C5—C6—C7	1.1 (4)	C13—C14—C15—C16	−0.6 (5)
N3—C5—C6—C7	−178.9 (3)	C14—C15—C16—C17	2.2 (5)
C5—C6—C7—C8	−0.2 (5)	C15—C16—C17—C12	−1.7 (5)
C6—C7—C8—C9	−0.3 (5)	C13—C12—C17—C16	−0.3 (4)
C7—C8—C9—C10	−0.1 (5)	C11—C12—C17—C16	179.1 (3)

### Hydrogen-bond geometry (Å, °)

**Table:**

*D*—H···*A*	*D*—H	H···*A*	*D*···*A*	*D*—H···*A*
O4—H4···O3^i^	0.82	1.84	2.654 (3)	175.
N3—H3···O1	0.86	1.97	2.685 (3)	140.
N1—H1···O3	0.86	1.99	2.631 (3)	131.
N1—H1···O2	0.86	1.91	2.568 (3)	132.

Symmetry codes: (i) −*x*+1, −*y*+2, −*z*+1.
